# User-centered design of contingency management for implementation in opioid treatment programs: a qualitative study

**DOI:** 10.1186/s12913-019-4308-6

**Published:** 2019-07-09

**Authors:** Sara J. Becker, Kelli Scott, Cara M. Murphy, Melissa Pielech, Samantha A. Moul, Kimberly R. Yap, Bryan R. Garner

**Affiliations:** 1 Center for Alcohol and Addiction Studies, Brown University School of Public Health, Box G-S121-5, Brown University, Providence, RI 02912 USA; 2 RTI International, 3040 E. Cornwallis Rd., Research Triangle Park, NC 27709 USA

**Keywords:** Opioid, Contingency management, Implementation science, User-centered design

## Abstract

**Background:**

Contingency management (CM) is one of the only behavioral interventions shown to be effective for the treatment of opioid use disorders when delivered alone and in combination with pharmacotherapy. Despite extensive empirical support, uptake of CM in community settings remains abysmally low. The current study applied user-centered design principles to gather qualitative data on familiarity with CM, current clinical practice, and preferences regarding the implementation of CM in community-based opioid treatment programs.

**Methods:**

Participants were 21 leaders and 22 front-line counselors from 11 community-based opioid treatment programs. Semi-structured interviews were about 45 min long. Transcripts from each interview were coded by independent raters and analyzed using a reflexive team approach. Frequencies of responses were tallied, and queries were run in NVivo to identify exemplar quotes for each code.

**Results:**

Results indicated low familiarity with CM, with less than half of the respondents defining CM correctly and over 40% of respondents declining to answer/ did not know. Abstinence was the most commonly recommended CM target, yet over 70% of respondents indicated that urine screens only occurred monthly. Attendance was also a popular recommendation, with respondents suggesting a range of possible indices including counseling, dosing, and/or case management sessions. Regarding the ideal role to administer CM prizes, program directors and supervisors were most commonly recommended, closely followed by front-line counselors. The most commonly suggested strategies to afford CM incentives included soliciting community donations and offering non-financial incentives.

**Conclusions:**

User design principles to understand workflow constraints, target user needs, and simplify the intervention guided this qualitative investigation of CM implementation in opioid treatment programs. Findings highlighted the potential value of flexible, organization-specific definitions of CM attendance and non-financial incentives, as well as active involvement of clinical leaders and supervisors to promote buy in among staff/patients. Respondents were generally optimistic about their ability to fundraise or solicit donations to overcome cost-related barriers of CM. Implications for CM implementation strategies, including the use of targeted leadership coaching focused on sustainability, are explored.

**Electronic supplementary material:**

The online version of this article (10.1186/s12913-019-4308-6) contains supplementary material, which is available to authorized users.

## Background

Opioid-use disorder-related overdoses and deaths have been declared a public health emergency in the United States [1], underscoring a dire need to increase access to evidence-based treatment in community settings. Contingency management (CM) is one of the only behavioral interventions that has been shown to be effective for the treatment of opioid use disorder [2, 3]. CM has demonstrated effectiveness both when delivered alone [4] and as a component of medication-assisted treatment [5]. Furthermore, when compared head-to-head versus other psychosocial interventions (i.e., cognitive behavioral therapy) as a component of medication-assisted treatment, receipt of CM is associated with superior rates of abstinence [6]. CM is based on principles of behavior analysis and involves consistent reinforcement of specific treatment-related goals via motivational incentives (e.g., prizes or vouchers) [7]. Over 100 randomized clinical trials have established the efficacy of CM (see 8), including a series of trials demonstrating that CM is effective when delivered by front-line treatment staff in opioid treatment programs [8, 9]. Most encouraging, a meta-analytic review found that adding CM to methadone treatment was associated with very large increases in abstinence rates (*r* = .56, d = 1.34) [5], suggesting that CM is ideally suited for implementation in opioid treatment programs (OTPs) that provide methadone.

Despite its extensive empirical support, uptake of CM in OTPs remains abysmally low. Surveys of the workforce in OTPs and other clinical settings treating patients with opioid use disorders suggest that most front-line treatment staff have never heard of CM and as few as 10% actually utilize it [10]. Commonly cited barriers to the utilization of CM include: financial costs associated with providing incentives; time required to stock prize cabinets; philosophical objections to rewarding patients; and limited knowledge of behavioral reinforcement principles in general or CM in particular [7, 10, 11]. Even when purveyors of CM have been able to overcome these barriers and integrate CM into treatment settings, sustainability has been a challenge. In a National Institute on Drug Abuse Clinical Trials Network study across six community OTPs, CM was implemented successfully, but only 12% of programs were able to sustain CM after removal of active support [12].

The challenges implementing and sustaining CM in community settings attest to the need for collaborative, user-centered design approaches with OTPs. Specifically, there is a need to obtain input from the target users of CM - front-line treatment counselors and leaders at OTPs - as to how to best design CM interventions for sustainability within their usual workflow. As described by Lyon and Keorner [13], three key principles of user-centered design for psychosocial interventions include: careful identification of intervention end users and their needs; simplification of existing intervention parameters and procedures to promote viable uptake; and consideration of system constraints to ensure that the end product fits the needs of the target end users. Guided by these principles, the overarching objective of the current study was to gather user feedback from both counselors and leaders at OTPs about their ideal CM design features, given their typical workflow and system constraints. Specific aims of the study were to elicit user preferences on the following CM design features: a) who should deliver CM (i.e., which staff would be best situated to deliver CM incentives), b) what CM should target (i.e., ideal behavioral targets to reinforce), and c) how CM prizes should be sourced (i.e., ideas for non-financial incentives and strategies to source prizes). The long-term goal of the current study was to inform the CM intervention design features used in a cluster randomized implementation trial with 30 OTPs throughout New England.

## Methods

### Recruitment and participants

Recruitment for this study occurred throughout the state of Rhode Island. The focus on Rhode Island reflected the funding for this study, which was designed to enhance research in Institutional Development Award eligible states. At the time of study initiation, there were 13 OTPs in Rhode Island. The President, Chief Executive Officer, or Director of the 13 OTPs were contacted by research staff and asked if they would like to nominate front-line counselors and leaders to participate. Eleven (85%) of the OTPs ultimately agreed to enroll in the study. Each OTP was allowed to select up to four staff (two leaders and two front-line treatment counselors) to participate. To be eligible for the study, leaders needed to be employed at least 6 months and have responsibility for supervising front-line staff. Front-line treatment counselors needed to be employed at least 3 months, have an active case load, and be responsible for providing psychosocial social support to patients receiving services in OTPs. One of the 11 sites only nominated one leader: in total, 21 leaders and 22 front-line counselors enrolled for a total of 43 recruited participants. Table 1 presents the demographics of the final sample of leaders and counselors, which was predominantly female (72%), White (93%), and bachelor’s level (42%). Average service tenure of participants at their current OTP was just under 5.0 years, with a wide range from 3.5 months to 41 years.

**Table 1:** Socio-Demographic Characteristics of Participants (*N* = 43)

	*N* (%) or *M* (SD)
Role in Opioid Treatment Program	
Leader	21 (49%)
Counselor	22 (51%)
Age (Years)	40.2 (14.4)
Biological Sex	
Female	31 (72%)
Male	8 (19%)
Other/ Prefer Not to Answer	4 (8%)
Race	
White	40 (93%)
Biracial or Multiracial	2 (5%)
Other	1 (2%)
Education	
High School Diploma	2 (5%)
Trade or Technical School	1 (2%)
Some College	8 (19%)
Bachelor’s Degree	18 (42%)
Some Graduate School	5 (12%)
Master’s Degree	9 (21%)
Experience at Opioid Treatment Program (Years)	4.8 (6.5)

### Procedures

All procedures were approved by the Institutional Review Boards at both Brown University and Miriam Hospital, which is affiliated with the Alpert Medical School of Brown University. Due to the minimal risk associated with participation, the study was granted waiver of documentation of consent. Prior to enrollment, interested counselors and leaders were provided with a research information letter, which described the study protocol in depth. Based upon this letter, counselors and leaders decided whether to provide verbal consent. Counselors and leaders were assured that their responses would be kept confidential (e.g., transcribed interviews would only be linked to a study identification number and would not contain names), that their feedback would not be shared with their employer, and that their decision whether or not to participate would not affect their employment in any way. In addition, counselors and leaders were assured that they could refuse to answer any questions or choose to withdraw from the study at any time.

### Qualitative interviews

The qualitative interview guide was developed by the research team to evaluate OTP leaders’ and treatment counselors’ experience with and preferences for CM (Additional file 1). Interviews were about 45 min long (range from 21 to 78 min), semi-structured, and completed by two trained postdoctoral research fellows and two research assistants (one Bachelor’s level and one Master’s level). Interview questions aimed to evaluate both current practice at each program site as well as preferences regarding the design and implementation of CM. Current practice questions included inquiries regarding typical caseload, typical patient contacts, and staff and leader receptiveness and reactions to new programs. Participants were also asked about their familiarity with CM and their perceptions of the elements of an ideal CM program.

The questions about CM design considerations were of particular interest for this study given the central goal of identifying who would be best suited to administer CM prize draws, ideal behavioral targets to incentivize for patients, and potential strategies to ensure affordability of incentives. Participants were asked by interviewers to provide detail regarding each of these CM design considerations. Each interview was conducted either over the phone or in person at the OTP with the audio of the interview recorded. All interviews were transcribed by an independent company upon completion of data collection. Transcripts were cleaned to assure they did not contain any potentially identifying information (e.g., participant name, participant organization) prior to analysis. Participants were assured that audio recordings would be destroyed 6 months after study completion. Front-line counselors and leaders received $100 cash for completing the interview, which was commensurate with other studies of clinical staff approved by the Brown University and Miriam Hospital Institutional Review Boards.

### Qualitative analysis

Consistent with the goals of the interview and structure of the interview guide, several a priori major themes were identified: familiarity with CM; ideal target behavior for CM; ideal staff to deliver CM; and affordability of incentives. These major themes were divided into subordinate themes using principles of thematic analysis [14].

Thematic analysis proceeded in an iterative process involving four coders (the principal investigator, a postdoctoral research fellow, and two research assistants) using a reflexive team analysis approach. [15, 16]. The goal of this method was to enable a team-based evaluation of subordinate themes across the specific CM design considerations. First, the coding team jointly developed a preliminary qualitative coding dictionary to guide formal coding processes. Next, each member of the coding team independently reviewed the same three transcripts and identified codes. The coding team then met to achieve consensus on the coding dictionary, including formal definitions for each major and subordinate theme identified. Following coding dictionary development, the postdoctoral fellow and two research assistants independently coded two additional transcripts to make final consensus-driven refinements to the data dictionary and to optimize initial inter-rater reliability. After the coding dictionary was finalized, the trained research assistants independently completed coding on all transcripts, with each research assistant coding approximately 19 transcripts (total independent *N* = 38), using NVivo version 12.0 qualitative coding software [17]. Emergent themes were added to the coding dictionary throughout the coding process to ensure all themes were captured. The postdoctoral research fellow served as a secondary coder for 20% of the completed interviews. Each of these 20% of transcripts were evaluated to identify potential disagreements, with meetings held weekly with the research assistants to facilitate consensus coding. In line with the reflexive team approach [11, 12], disagreements in coding were resolved via in-depth discussion and decision-making among the coders until 100% consensus was obtained.

Once codes were assigned to all transcripts, queries were run in NVivo to tally frequency counts of the most common themes in responses to interview questions regarding current familiarity with CM; current clinical workflow and system constraints; and ideal CM design considerations (e.g., who would administer CM, what behavior should be targeted, and strategies, to ensure affordability of incentives). Because our objective was to elicit provider preferences, we assigned numeric frequencies to enhance the precision of results reporting, to facilitate pattern recognition, to provide systematic evidence of the diversity of respondent preferences, and to document which design features were the most popular among respondents [18]. As a final step, targeted queries were run in NVivo to identify exemplar quotes for each subordinate theme.

## Results

The primary goal of the current analysis was to apply user-centered design principles to solicit feedback from front-line treatment counselors and leaders about their ideal CM design features, given their current workflow and system constraints. Table 2 presents frequency counts of themes related to current workflow and Table 3 presents frequency counts of the themes related to ideal CM design. We examine each dimension and provide illustrative quotes in the sections below.

**Table 2:** Frequencies of Codes Related to Current Workflow (*N* = 43)

Code	Number of Respondents
Contingency management familiarity	
Correct definition	18
Vague definition about incentives	7
Incorrect definition / did not know / did not respond	18
Frequency of urine screens	
Monthly	31
2–3 times per month	4
Weekly	4
Not sure / not discussed	4
Frequency of counseling	
Monthly	25
2–3 times per month	6
Weekly	5
Not sure / not discussed	7

**Table 3:** Frequencies of Codes Related to Ideal Contingency Management Design Features (*N* = 43)

Code	Number of Respondents
Behavioral Targets	
Abstinence from opioids or all drugs	29
Attendance at counseling sessions	25
Attendance at dosing sessions	16
Attendance at sessions made by case management	8
Who will Administer	
Program director or management	10
Clinical supervisor	10
Primary counselor	9
Front desk staff	3
Nursing staff	1
Non-Financial Incentives	
Take home doses	17
Certificate or reward	9
Less frequent counseling sessions	6
Preferential parking	4
Line jump or treatment pass	3
Extended dosing hours	3
Less frequent urine screens	1
Ways to Obtain Incentives	
Community donations	23
Grant writing	8
Donations from staff	4
Fundraising	3

### Current operations: familiarity with CM and system constraints

At the start of the qualitative interviews, the 43 counselors and leaders were asked to define CM in their own words (see Table 1). We coded a definition as correct if it referenced rewarding patients or providing incentives for meeting treatment goals. Less than half of the respondents (*n* = 18, 42%) defined CM correctly. An equal number of respondents (*n* = 18, 42%) said that they did not know or preferred to not answer the question. Another seven respondents (16%) provided a vague definition that made reference to “reinforcement,” “motivating clients,” or giving “incentives,” but did not specify meeting goals. Thus, familiarity with CM was relatively low in the current sample.

A key aspect of user-centered design principles is understanding the current needs and system constraints of the target user. Consistent with this approach, respondents described the typical workflow at their organizations and the frequency with which patients received dosing, urine screens, and counseling sessions. Because all of the participating sites provided methadone, respondents uniformly reported that dosing was provided daily. Over 70% of respondents (*n* = 31) reported that, on average, patients received urine screens monthly, in line with state regulations. Respondents also reported that urine screens were typically sent off site for testing, with delays ranging from two to 7 days to obtain results. The frequency of counseling sessions was similar with most respondents (*n* = 25) reporting that sessions were provided monthly. It is important to note that these estimates reflected averages: the vast majority of respondents reported that new patients (i.e., those in the first 4–8 weeks of treatment) and those identified as “at risk” (i.e., those with poor compliance or positive urine screens) received counseling sessions and urine screens more often, whereas patients with longer tenure received less frequent contact.

### Dimension 1: ideal behavioral targets

After examining current operations and system constraints, focal questions shifted to examining ideal CM design features. The first feature explored was the ideal behavioral target. Counselors and leaders spontaneously offered several distinct potential behavioral targets to reinforce via CM. The most frequently mentioned target (see Table 2) was abstinence, closely followed by attendance at counseling sessions. Of note, respondents rarely suggested only one behavioral target: in fact, over 70% of respondents that suggested abstinence also recommended at least one attendance goal.

Most respondents (*n* = 29) commented that abstinence, confirmed via “negative tox screens,” would be the ideal target because it is “the biggest struggle” for patients. Among those suggesting abstinence, six specifically said that patients should receive incentives for being negative from opioids, even if they tested positive for other drugs. Of these, half (*n* = 3) felt that incentives should start by targeting abstinence from heroin and fentanyl because of the risks of overdose. For instance, one counselor said “there’s say someone that is using heroin for 20 years straight and, literally, you do the contingency with them and they’re doing cocaine but they stopped opioids, I still feel like they need to be incentivized for that.” By contrast, five respondents felt that “total abstinence” from alcohol and all other drugs should be the goal.

The next most frequently recommended behavioral target (*n* = 25) was attendance at counseling sessions. Multiple respondents shared their perspective that counseling sessions are essential to teach patients the skills needed to maintain sobriety in the longer-term. One leader commented, “in theory if a person’s keeping their counseling appointments, they would be getting the support they need to help them achieve abstinence,” while another said “if you don’t come to treatment, you are going to relapse.” A few counselors felt that attendance at counseling was an ideal target, because their organizations often struggled with “people not keeping their appointments.”

The third most commonly suggested target (*n* = 16) was “dosing attendance” and/or compliance with dosing. Common reasons for encouraging daily dosing included building a sense of stability, fostering a sense of engagement with the program, and ensuring a therapeutic dose. For instance, one leader posited:

“Dose attendance… that would be helpful… especially because maintaining that structure can help them with the medication actually being effective, and then increasing their stability… Whether it’s if they’re here they’d be more likely to keep their counseling appointments. If they’re up and they’re in a routine they’re more likely to develop stability in other parts of their life.”

Finally, eight respondents suggested that follow through with case management services would be a valuable target. One counselor specifically noted that they “refer a lot of patients” to outside services and that they frequently have to ask patients for status updates. Examples of potential outside services to incentivize included: “get engaged with mental health,” “fill out that Social Security application,” “meet with the job coach,” “get a primary care doctor,” “have a full dentist exam,” and “go to one outside recovery group.”

### Dimension 2: ideal role to administer CM

The second dimension examined was the ideal role to administer CM prize draws at the opioid treatment programs. Respondents suggested that CM prize draws could be administered by a range of different employees. The two most commonly recommended roles were program directors (*n* = 10) and clinical supervisors (*n* = 10), though of note, many respondents suggested these two roles as interchangeable. The primary reasons for suggesting leadership positions such as directors and clinical supervisors included: a) ability of leadership to be objective when making decisions about prizes; b) concern that counselors might appear biased if they administered incentives; c) desire to have organization leadership more intimately involved in clinical services; d) lower case load of leaders relative to counselors; and e) ease of getting leadership approval to administer prizes with monetary value. To illustrate, one leader said:

“I think primarily a supervisor or a director and only that because they can look at holistically to say, oh yes, they’ve met the criteria for the incentive, you’re right. It also pulls them into loops; they know exactly what’s happening. I think that the clinicians tend to be discretionary based on clients that they like more…”.

Meanwhile, another counselor posited that patients would feel more motivated if they received prizes from a leader: “I guess the program director, because I think that when they feel supported by the top person in their site, they feel like they have everybody on board.” Finally, a few counselors commented that leaders had “limited caseload” and that having a leader designated would “take a little bit off the rest of us that are down here doing the daily contact.”

Primary counselors were also nominated frequently (*n* = 9). The primary reason for recommending counselors was their clinical rapport with patients. This theme was echoed in quotes such as, “they’re probably closest to the patients,” “that’s the relationship between the patient and clinician,” and “they’re the person working with them.” Respondents also felt that counselors would be able to uniquely customize feedback during CM sessions, as illustrated by the following quote: “they [prize draw sessions] should be more individualized and the counselor is going to know their caseload and what they need.”

Less frequently nominated individuals included front desk staff and nurses. Three respondents felt that front desk staffs could administer prize draws because “they know all the patients.” Of note, all three of these respondents recommended a program director or supervisor as the preferred option. Finally, one counselor suggested a nurse or nurse case manager, because nurses “see the patients daily” and have “a discrete area where the patient could go.”

### Dimension 3: ideal strategies used to ensure affordability of CM incentives

Respondents were also encouraged to consider strategies to afford CM incentives. Such strategies fell into two broad themes: ideas to source incentives and ideas around non-financial incentives. Regarding the sourcing of incentives, by far the most common idea was to request community donations (*n* = 23) of goods (e.g., clothes, toiletries, meals, narcan kits) and services (e.g., job coaching, haircut). The majority of respondents spoke positively about community donations, noting that it would be “absolutely doable” and would be a natural outgrowth of “good relationships” and existing “community outreach.” By contrast, a few respondents raised concerns that stigma around opioid addiction might make it difficult to obtain donations. For instance, one leader said “I would like to think that certain agencies wouldn’t have a problem being attached to it, but again, I don’t know if they want to give to a methadone program.” Several respondents explicitly suggested that leadership should be responsible for soliciting donations, noting that it would foster institutional buy in and strong community partnerships.

The next most common idea was grant writing (*n* = 8). Respondents expressed optimism that their organizations could obtain state, Medicaid, or private foundation funding to cover incentive costs. Of note, all of the respondents that mentioned grant writing reported that they had experience obtaining grants and/or dedicated staff (e.g., “corporate people,” “people [whose] job it is to find that money”) tasked with grant writing for special programs.

Other less common ideas included having staff personally provide donations or hold fundraisers. Four respondents suggested that staff could donate their own time, resources, or services to get things started. One leader noted that starting with staff donations might be necessary because, “We have a very tight budget and that’s why a lot of things that we have to implement, we have to come out of out our own pocket for.” Meanwhile, three respondents suggested that staff could lead fundraisers such as car washes, raffles, and community events.

With regards to non-financial incentives, respondents generated a number of ideas of incentives that would be appealing to patients that would not cost money. Seventeen respondents mentioned take-home doses as highly motivating for patients, though a consistent theme was that such doses were heavily regulated and therefore difficult to provide to new patients. Another popular idea (*n* = 9) was to provide positive recognition via a certificate, trophy/token, award ceremony, or client appreciation event. One counselor conjectured that “even though it’s a piece of paper, that’s a sense of accomplishment that maybe they’ve never gotten before.” Other ideas included incentives to make the process of treatment more convenient for patients such as front of the line passes, preferential parking, a free pass or ability to miss a counseling session, and extended hours for dosing. One counselor became very excited when thinking about non-financial incentives and noted, “This is what’s cool about contingency-based work. We could be really inventive. We could really brainstorm some great ideas.”

## Discussion

This qualitative study solicited input from front-line treatment counselors and leaders to inform the CM intervention design features used in a cluster randomized implementation trial with 30 opioid treatment programs throughout New England. Following principles of user-center design [10], we collected information from respondents in the settings that will ultimately be implementing CM around several areas: what should be targeted; who should implement CM; and how incentives could be kept affordable. User-centered design takes into consideration factors of the intended setting that may constrain how an evidence-based treatment is used, including the target audience, frequency of intervention delivery, budget, and operating costs [13]. Enlisting end users in CM intervention design has been recommended as a way to increase the likelihood that counselors’ concerns are addressed and that intervention parameters are consistent with constraints in the environments where counselors work [14]. In harmony with prior work [10], rates of CM familiarity were low across the 11 OTPs, highlighting significant opportunities to enhance the implementation of CM in this high need setting.

With regards to the optimal behavioral target to reinforce, abstinence and attendance – both of which have been shown to be effective targets in meta-analytic reviews [19] - were reported most frequently. However, consideration of the typical workflow at the OTPs raised several constraints that could pose challenges to the use of abstinence as a behavioral target for CM. For instance, over 80% of respondents reported that urine screens were administered monthly at their sites, and multiple respondents noted that urine screens were sent off site for testing. Inability to assess the behavioral target at least weekly [5] or to provide immediate feedback to patients [19] have both been found to reduce treatment effects. Thus, a significant change in the typical workflow and significant expenditures on more frequent rapid on-site testing would be needed for regular monitoring of this target.

A narrow definition of treatment attendance focused solely on counseling sessions could also present challenges for sites, particularly as the majority of respondents indicated that patients were generally seen weekly for the first 4–8 weeks of counseling and then seen monthly. One viable solution within the constraints noted in the interviews could be to target some measure of attendance, flexibly defined based on the preferences of the organization, which could include attending individual or group counseling sessions, dosing sessions, case management sessions, and/or case management referrals. Targeting attendance is likely to be a particularly viable strategy for new patients or those identified as at risk, since these patients are likely to receive the most frequent clinical contact. Notably, a prior study found that patients receiving CM targeting attendance as an adjunct to treatment had higher treatment utilization and estimated reimbursement rates over 1.5 times greater than patients receiving treatment as usual without CM [16]. Accordingly, reinforcing attendance could serve to increase revenue from billable clinical services, which could help to offset the costs of CM.

Another key user-centered design principle for developing and implementing an intervention [13] is clear identification of the target end user. Somewhat unexpectedly, a large number of respondents thought that the best person to administer CM prize draws would be someone in a supervisory/managerial role, though counselors were suggested as a close second option. When queried as to why, one reason that emerged was that individual counselors might be more flexible and “discretionary” with providing incentives, whereas leadership would exercise greater objectivity. In addition, counselors reported that they had a larger caseload of patients relative to leadership, implying time constraints of current clinical responsibilities may dissuade them from adopting the practice. Such concerns could reflect limited understanding of behavioral reinforcement principles and a misperception that CM is time-intensive and distinct from typical clinical care. Increased knowledge and readiness to adopt CM, as well as positive perceptions regarding the cost, feasibility, and sustainability of CM have been shown following CM training [20]. Providing specialized CM training to counselors could potentially help to clear up any misconceptions about CM as a therapeutic approach: specifically, a clear understanding of CM could potentially increase confidence in one’s ability to be objective when delivering incentives and could allow counselors to feel more adequately prepared to adopt CM within the current demands of their positions. Participants’ responses also highlighted the need to carefully consider ways to simplify CM procedures, such as reducing the number of steps, in order to decrease the cognitive load required [13]. While it is possible that the aforementioned strategies could serve to ameliorate concerns about CM among counselors, it is equally plausible that implementing CM with site leaders and directors represents a novel strategy for CM delivery worthy of further investigation.

Finally, the interviews examined strategies to source high-salience, affordable reinforcers that can successfully compete with reinforcement from using opioids [21]. Such strategies are imperative considering that the disconnect between research and practice costs has been noted as a barrier to implementation [7] and costs (both indirect costs of staff time and direct costs of incentives and toxicology screens) have been found to lead sites to decide against implementing CM [21, 22]. Respondents generated a number of ideas of non-financial incentives that would be appealing to patients beyond take-home doses, such as recognition via a certificate, trophy/token, or an award / appreciation ceremony. These ideas were consistent with recommendations in the CM literature to gather information from organizations on what patients value and offer a mix of social (e.g., praise, reinforcement) and material (e.g., prizes) incentives [23]. When considering ways to source other incentives, more than half of the respondents suggested requesting community donations. Prior research suggests that this approach could be feasible for community-based treatment programs; a study of a voucher-based CM program found that more than a third of local companies and public organizations who were asked to donate specific goods or services (e.g., newspapers or magazines, public transportation, tickets to cinemas, sporting events, museums, or other leisure services) provided donations [22]. Taken together, our results indicate that front-line treatment counselors and leaders were optimistic about their ability to source incentives and did not view the cost as an unsurmountable impediment to CM delivery. Moreover, multiple respondents suggested that responsibility for sourcing incentives should rest with organizational leadership, highlighting the value of implementation strategies directed towards leaders and supervisors.

## Limitations

The present study had several limitations that are important to acknowledge. First, the fact that each organization was allowed to nominate two leaders and two counselors for participation could have introduced selection bias. It is possible that our results reflect the perspectives of the most favored or experienced CM staff, and not necessarily the perspectives of all employees at OTPs. Second, the lack of diversity among counselors and leaders was a concern. It is important to note that the participant demographics points towards a broader challenge within the addiction workforce, which has been found to be predominantly White and female [23]. Finally, though consistent with prior surveys of the addiction workforce (24), the relatively low level of familiarity with CM among respondents could have limited the scope of responses. Participants with higher levels of familiarity might have generated additional novel ideas about CM targets, delivery, and affordability. Notwithstanding these limitations, the application of user-centered design principles to inform subsequent intervention delivery represented a novel strategy that provided input on CM design features best aligned with usual services in opioid treatment programs.

## Conclusions

Overall, the data from the present investigation suggested that ideal CM design features at participating OTPs would include supervisory/leadership staff or counselors administering CM prize draws targeting attendance to those patients most conducive to frequent monitoring (i.e., new patients or those identified as at high risk). Respondents had numerous ideas for no-cost incentives that patients would value and believed that donations from community businesses would be a viable way for organization to source additional incentives. When considering these preferences in the context of system constraints at the OTPs, results highlighted several considerations for researchers and clinical programs seeking to design CM for sustainability, which will guide the CM parameters used in a forthcoming cluster randomized trial with 30 OTPs. Specifically, the CM protocol used in the upcoming trial will have the following parameters: a) each OTP will develop an organization-specific, flexible definition of patient attendance (encompassing clinical services that are most reimbursable and most meaningful to each organization); b) CM will be targeted to new patients within 1 month of admission, as new patients were the most likely to be monitored at least weekly across the participating OTPs; c) CM will be delivered by front-line counselors, who will receive specialized training to increase knowledge, confidence, and readiness for adoption of CM; and d) organizational leadership will be actively involved in all aspects of CM training to ensure sufficient buy in and support for CM implementation. Results of the current study further suggested that implementation strategies directed towards OTPs would benefit from engaging organizational leaders and clinical supervisors in planning for long-term sustainability, given the need for organizational leaders to support the provision of meaningful non-financial incentives and the solicitation of donations. Thus, the forthcoming cluster randomized trial will empirically evaluate whether Implementation Sustainment Facilitation, an implementation strategy that helps organizational leaders and counselors to plan for long term sustainability (25), can improve the implementation and sustainment of CM in OTPs relative to comprehensive training without sustainment-focused support.

## Additional file

Additional file 1: Qualitative Interview Guide. This guide contains the qualitative questions developed for the current study. (DOCX 28 kb)

## Data Availability

De-identified data are available from the corresponding author on reasonable request.

## Abbreviations

CM: Contingency management
OTP: Opioid treatment program
