# Supplementary material for: User-centered design of contingency management for implementation in opioid treatment programs: a qualitative study
Source: BMC Health Serv Res. 2019 Jul 9;19:466. doi: 10.1186/s12913-019-4308-6 (PMC6617614; doi:10.1186/s12913-019-4308-6)
Supplement: Supplementary file 1 — Qualitative Interview Guide. This guide contains the qualitative questions developed for the current study. (DOCX 28 kb) [file 12913_2019_4308_MOESM1_ESM.docx]

**Contingency Management in Combination with Medication-Assisted Treatment for Opioid Use Disorders**

**INTERVIEW DETAILS**

**Date: _____________** *(MM/DD/YYYY)*

**Interviewer’s initials: _____________**

**Participant ID: _____________**

**Was potential subject provided the research information letter?**

- - **Yes**
  - **No**

**Did potential subject participate in the verbal consent process and agree to be in the study?**

- - **Yes**
  - **No**

**Does potential subject meet the eligibility criteria for this study?**

- - **Yes**
  - **No**

*Thank you for agreeing to participate in this study. This interview is completely voluntary, so please feel free to skip any question or stop the interview at any time. Please also feel free to ask questions at any time if anything is unclear.*

**INTERVIEW PROTOCOL**

**INTRO SCRIPT: *Thank you for taking the time to meet with today. This interview will last about 45 minutes. I will start by asking you some questions about how [your agency] approaches the implementation of new practices, and then I will ask your opinion about a specific treatment approach called contingency management.***

***Do I have your permission to begin recording*? [START RECORDING]**

***This is [interviewer name] at [agency] on [date] with [participant number].***

*Note: This guide has been organized such that the questions that were the focus of the contingency management design analysis are in bold for ease of identification.*

**Section 1**

*To begin, I have a few questions about your professional background and [your organization].*

***Note for the Interviewer***: Portions of questions following e.g. should only be used if the participant does not spontaneously offer content. The e.g. is intended to illustrate so that the interviewer understands the question and can provide clarification if needed. The goal is not to read these options but rather to give the participant space to respond.

**Introduction / Background**

1. What is your title and role within [agency/facility name]?
   1. What does that entail?

**Domain 1: Typical Workflow / Case Load**

1. **Tell me a little bit about typical case load at your program.**
   1. **How often do you see patients? Is this the same for new versus established patients? (Probe if needed: How do you define a “new” patient?)**
   2. **Do you see all patients the same frequency or does it vary by patient? If it varies, how?**
2. **Tell me a little bit about typical patient flow in [your organization].**
   1. About how many new patients would you estimate typically enroll on a monthly basis?
   2. **What is the typical course of treatment once a patient enrolls? How often do patients receive medication? Attend counseling sessions? Receive other services?**
3. **Tell me about the current urine screening process at [your agency].**
   1. **How often are patients screened? How do the requirements change as the patient progresses in the program (e.g., less frequent attendance or screening required, take-home doses allowed)?**
   2. How is it decided when/whether a patient will be screened?
   3. Who is responsible for screening these patients?
   4. Is the patient monitored/supervised during the screen? If so, how?
   5. **How quickly does [your agency] get the results of the test? Does [your agency] have the ability to give rapid results?**
   6. When, how, and by whom is the patient informed of the results?
4. In your opinion, how receptive are staff in [your organization] to new and innovative programs and practices? (Probe: Can you tell me more about that/about a time when a new program or practice was implemented here? How did staff react?)
5. In your opinion, do you think the leadership of (or other leadership in) [your organization] is supportive of new or innovative programs? Why/why not?

**Domain 2: CM Familiarity**

*Thank you for answering those questions. Next, I’d like to get some information from you about your opinions of a specific type of treatment called contingency management (also known as motivational incentives).*

1. **From your perspective, how would you define contingency management? If you’re not sure how to define it that’s okay.**

[If not a clear answer/unsure provide a definition]. *Contingency management is a behavioral treatment that provides patients with motivational incentives for pre-specified behavioral targets. The general principle is to provide positive incentives that compete with the choice to continue to use drugs. Typically, individuals in substance use disorder treatment will receive incentives for certain behaviors (e.g., attendance, abstinence) to help motivate them to refrain from using drugs.*

1. Are you aware of programs or providers using contingency management?
2. Have you personally ever used contingency management?
3. Some providers may not be comfortable with providing incentives as a way to motivate patients to refrain from using drugs. For you personally, how comfortable or uncomfortable would you be with [your agency] providing incentives? Why?
   1. If a patient had a urine screen that was negative for opioids but had a positive screen for another drug, would you feel comfortable giving the patient an incentive? Why or why not?
4. In your opinion, what might be some of the benefits of implementing contingency management in [your agency]?
5. In your opinion, what might be some of the barriers that would impact the implementation of contingency management at [your facility]?

**Domain 3: CM Intervention Design**

1. **If you could design a new contingency management program in [your agency], what would be some of the key elements?**
   1. **Can you think of any non-financial incentives that might help motivate patients at [your agency]?**

**[If not clear / unsure can define]: *Non-financial incentives are prizes that would not cost you anything to provide.***

**Are there any non-financial incentives specific to [your agency] that you think could help motivate patients (e.g., parking, dosing schedule, going to the front of the line)?**

- 1. **Can you think of any ways that [your agency] might be able to source incentives to provide to patients that would not cost you any money (e.g., gifts, grants, donations)?**
  2. **What do you think would be most important behavioral target to incentivize for your patients (e.g., attendance, abstinence from opioids, abstinence from all drugs)?**
  3. **If [your agency] designated a specific position / role to administer the prize draws, who do you would be the best person to do that? Why?**

1. One decision we are trying to make is the best patient age range to serve. Does [your organization] provide services for youth under 18? If not, where would you typically refer a parent or youth under the age of 17 looking for services?

**Domain 4: CM Training Design**

1) In your opinion, what would be the key elements of a training program to help staff learn

contingency management?

- 1. Many agencies rely on didactic training like a workshop or seminar. What types of support might you like in addition to didactic training?
  2. Would you like ongoing support? If so, like what?
  3. What do you think might help motivate staff to learn contingency management?

2) Some people say that receiving performance feedback on their delivery of a new model

can be helpful after training. If [your organization] were to provide you with feedback on

your delivery of contingency management, how would you prefer to receive it?

- 1. How would you most value feedback (e.g., chart review, audio recordings of sessions, rating sheets you fill out, rating sheets your patient fills out, etc)?
  2. From whom would you most value feedback (e.g., external expert, supervisor within the agency, colleague, etc.)?

**SCRIPT:** *Thank you, again, for your time today.*

*Do you have any other thoughts that you would like to share about contingency management, the process of adopting a new practice at [your agency], or any other topics that we’ve talked about today?*

**[STOP RECORDING]**

**SCRIPT:**

*To end, I have a few background questions.*

**Section 2**

**1. What is your age?** *[PPTAGE]*

___________ [minimum value = 18]

**2. What is your gender?** *[GEN]*

- Male
- Female
- Something else: __________ [OTH]
- DK/R

**3. Are you of Hispanic or Latino decent?** *[ETHNIC]*

- Yes
- No
- DK/R

**4. How would you describe your racial background?** *(Check only one). [RACE]*

- American Indian or Alaska Native
- Asian
- Black, African, Haitian, or Cape Verdean
- Native Hawaiian or other Pacific Islander
- White
- Mixed, bi-racial, or multi-racial
- Something else: __________ *[OTH]*
- DK/R

**5. What is the highest level of education that you have received?** *(Check only one).* *[EDUC]*

- Elementary or grade school
- Some high school
- Finished high school or GED
- Some college
- Trade or technical school
- College or university degree
- DK/R

**6. How many years have you been with your current agency/facility?** *[EXP]*

*____* years *____* months
